# Mechanism of Microwave Radiation-Induced Learning and Memory Impairment Based on Hippocampal Metabolomics

**DOI:** 10.3390/brainsci14050441

**Published:** 2024-04-29

**Authors:** Shuting Guan, Yu Xin, Ke Ren, Hui Wang, Ji Dong, Haoyu Wang, Jing Zhang, Xinping Xu, Binwei Yao, Li Zhao, Ruiyun Peng

**Affiliations:** Beijing Institute of Radiation Medicine, Beijing 100850, China; guanst59@163.com (S.G.); lillyliz@163.com (L.Z.)

**Keywords:** microwave radiation, hippocampus, learning and memory, non-targeted metabolomics

## Abstract

The brain is complex and metabolically active, and the detection of metabolites plays an important role in brain development and diseases. Currently, there is a lack of research on the metabolic spectrum changes in learning and memory impairment, and hippocampal damage induced by microwave radiation from the metabolic perspective. Aiming to provide sensitive indicators for microwave radiation-induced brain damage and establish a foundation for understanding its injury mechanisms, this study employed non-targeted metabolomics to investigate metabolic fluctuations and key metabolic pathway alterations in rats’ hippocampal tissue after microwave radiation. The memory and spatial exploration abilities of rats decreased after radiation. The postsynaptic densities were thickened in the MW group. The cholesterol sulfate, SM(d16:1/24:1(15Z)), and linoelaidylcarnitine were significantly increased after radiation, whereas etrahydrocorticosterone, L-phenylalanine, and histamine were significantly decreased after radiation. These metabolites were enriched in signaling pathways related to the inflammatory mediator regulation of transient receptor potential (TRP) channels, neuroactive ligand–receptor interaction, steroid hormone biosynthesis, and phenylalanine, tyrosine, and tryptophan biosynthesis. These findings indicate that microwave radiation causes spatial learning and memory dysfunction in rats and structural damage to hippocampal tissue.

## 1. Introduction

With the rapid development and widespread application of electromagnetic wave technology, electromagnetic radiation has become the fourth major environmental pollutant after “three wastes” pollution [1,2]. Microwaves with frequencies between 300 MHz and 300 GHz are the most widely used. People are increasingly exposed to microwave radiation environments, and its potential health hazards are receiving more attention [3]. Microwave radiation can cause damage to multiple systems and organs in the body, especially the nervous system [4]. Epidemiological studies have found that microwave radiation can cause neurasthenia, and there is a dose–effect relationship. With prolonged exposure, the frequency of symptoms such as headache, dizziness, and memory decline significantly increase [2,3]. The hippocampus is a key brain region closely related to learning and memory. Studies have found that microwave radiation can cause damage to hippocampal tissue, including neuronal degeneration, apoptosis, and necrosis, disrupted neurotransmitter release, mitochondrial swelling, fragmentation, and vacuolation [5,6,7,8]. Behavioral experiments related to hippocampal function, such as the Morris water maze and Novel Object Recognition test, have shown that rats exposed to microwave radiation have prolonged average escape latency and abnormal brain electrical activity, indicating a decline in learning and memory abilities, spatial memory abilities, and recognition memory abilities [9,10,11]. However, the sensitive indicators and mechanisms of injury remain to be elucidated.

The brain is complex and metabolically active, and the detection of metabolites plays an important role in brain development, diseases, etc. Various metabolites have been elucidated as biomarkers for understanding the pathogenesis of neurodegenerative diseases such as Alzheimer’s disease and Parkinson’s disease, and as intervention targets to adjust the risk and resilience of neurodegenerative diseases [12,13,14,15]. Neurodegenerative diseases are neurological disorders characterized by degenerative damage to neurons and the consequent loss of both structural and functional integrity of the nervous system. Common neurodegenerative diseases include Alzheimer’s disease, Parkinson’s disease, Huntington’s disease, and amyotrophic lateral sclerosis (ALS) [16]. Studies have shown that in Alzheimer’s disease patients and mouse models, hippocampal neuron axonal swelling and demyelination occur [17]. In Parkinson’s disease rodent models, there is a reduction in axonal length, decreased vesicle transport, and increased axonal degeneration [18]. ALS patients exhibit abnormal peripheral motor neuron axonal growth and transport function, leading to the degeneration of motor neurons [19]. Additionally, research indicates that Alzheimer’s disease model rodents display cognitive impairment in behavioral studies [20]. The effects of microwave radiation, such as impaired learning and memory and synaptic dysfunction, bear similarities to findings in various neurodegenerative disease studies, suggesting a potential correlation between microwave radiation and neurodegenerative diseases.

Currently, there is a lack of research on the metabolic spectrum changes in learning and memory impairment, and hippocampal damage induced by microwave radiation from the metabolic perspective. Our team has conducted extensive studies on microwave radiation at 2.856 GHz, and has discovered varying degrees of damage to the nervous system, reproductive system, and heart under such radiation. A mature rat model of learning and memory impairment has been established using this frequency range. Aiming to explore the metabolic spectrum changes in learning and memory impairment, and hippocampal damage induced by microwave radiation, this study found the metabolic fluctuations and key metabolic pathway changes in the hippocampal tissue of rats after 2.856 GHz microwave radiation using untargeted metabolomics, and analyzed their role in learning and memory impairment.

## 2. Materials and Methods

### 2.1. Animals and Treatment

Sixteen secondary male Wistar rats weighing 180 ± 20 g were purchased from Beijing Vital River Laboratory. They were housed in the animal center of the Beijing Institute of Radiation Medicine, with a room temperature of 25 °C, relative humidity of 60%, and a 12 h light/12 h dark cycle. The rats had free access to food and water, with three rats per cage. After 3 days of adaptive feeding, the rats were weighed, and then randomly divided into a Sham group and a Microwave Radiation (MW) group, with 8 rats in each group.

### 2.2. Microwave Exposure Method

**Figure 1:**
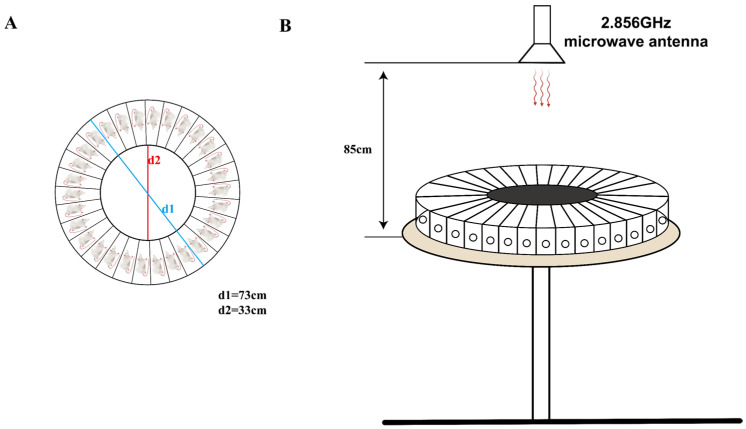
This is a schematic diagram of microwave radiation in rats. (**A**) Overhead view of the rat radiation box; (**B**) front view of microwave radiation.

The rats were irradiated with microwave radiation using a microwave radiation source from the Beijing Institute of Radiation Medicine. To reduce reflections, the radiation source was placed in an electromagnetic shielding room covered with conical microwave absorbers on the walls. The rat irradiation tray was made of organic glass, with a diameter of 73 cm, divided into 30 equally sized independent spaces by organic glass plates, and equipped with corresponding covers (Figure 1A). The irradiation tray with the rats was placed on the irradiation platform, and the platform was raised to a height of 85 cm from the radiation source (Figure 1B). During irradiation, the irradiation platform rotated uniformly. The rats were irradiated with microwaves at a frequency of 2.856 GHz, with an average power density of 30 mW/cm^2^, for 15 min each time, three times in total, with a 5 min interval between each time. The Sham group of rats underwent the same procedures as the MW group but without radiation.

### 2.3. Y-Maze Behavioral Test

Six hours after microwave radiation exposure, the Y-maze experiment was conducted on the rats. The three arms of the Y-maze device were labeled as the start arm, other arms, and novel arm. During the learning phase, the novel arm was closed, and the rats were placed facing the maze wall into the start arm. The rats were allowed to explore the maze for 10 min and then returned to their cage. One hour later, during the testing phase, the novel arm was opened, and the rats were placed facing the maze wall into the start arm to explore all arms for 5 min. The behavior of each rat during the test was recorded by a camera and analyzed using Anymaze animal behavior analysis software. After each rat completed the test, the maze was wiped clean with 75% ethanol [21].

### 2.4. Novel Object Recognition Test

On the day before radiation, rats were placed facing the walls of the maze and allowed to acclimate in one corner of the open field for five minutes each. Six hours after microwave radiation, rats underwent the Novel Object Recognition (NOR) test. During the learning phase, two identical rectangular prisms were placed in the open field, and each rat explored for 10 min, with exploration time recorded. One hour after the end of the learning phase, rats entered Test Phase I. One of the rectangular prisms in the open field was replaced with a cone of similar size. Rats were placed in the field and allowed to explore for 5 min each, with exploration time between different objects recorded. Twenty-four hours after the end of the learning phase, rats entered Test Phase II. The new object (cone) was replaced with another new object (cylinder), while the other object remained a rectangular prism. Each rat explored for 5 min, and exploration time between different objects was recorded. After each rat’s experiment, the bottom of the box was wiped with alcohol to prevent odor residue. The discrimination index (DI) was used to reflect the recognition ability of rats, where F represents the exploration time of familiar objects, N represents the exploration time of novel objects, and the calculation formula is $DI=N/\left(N+F\right)\times 100\%$.

### 2.5. Transmission Electron Microscopy

Six hours after microwave radiation exposure, the rats were anesthetized with 1% pentobarbital sodium (50 mg/kg) injected intraperitoneally, decapitated, and their brains were quickly removed. The hippocampal tissues were rapidly isolated, and 1 cm³ tissue blocks were sliced and fixed in 2.5% glutaraldehyde phosphate buffer, followed by fixation in 1% osmium tetroxide. Semi-thin sections of 1.5 μm were made and stained with hematoxylin-eosin staining (HE) for light microscopy observation and localization, and then ultra-thin sections of 70 nm were made and double-stained with acetic acid and lead nitrate [22]. The ultrastructure of hippocampal tissue synapses was observed by transmission electron microscopy. ImageJ software was used for quantitative analysis of neuronal ultrastructure. For morphometric analysis of synapses and mitochondrial cristae, the postsynaptic density thickness, mitochondrial cristae, and ratio of the mitochondrial inner membrane to outer membrane were measured using the Image J software (ImageJ 1.53e; Java 1.8.0_172).

### 2.6. Western Blot

Six hours after microwave radiation, hippocampal tissue was collected from rats. Approximately 10 mg of rat hippocampal tissue was placed into a 1.5 mL EP tube along with 3–5 small magnetic beads. Subsequently, 150 μL of tissue lysis buffer (with a 1:100 dilution of protease inhibitor) was added. The sample was homogenized using a homogenizer at 60 Hz for 4 cycles, with each cycle lasting 60 s and a 15 s interval between cycles. After homogenization, the sample was left to stand on ice for 10 min, followed by centrifugation at 12,000 rpm for 10 min at 4 °C. The supernatant was collected into a new EP tube, and the protein concentration was determined using the BCA method. Simple Western-Jess was utilized for Western blot analysis [23]. The samples and ladder were prepared using the standard pack provided by the manufacturer to achieve a final protein concentration of 0.2 μg/μL. The protein samples were denatured at 95 °C for 5 min and stored on ice. PSD95 was diluted 1:100 and GAPDH was diluted 1:500 using the Antibody Diluent provided in the kit. A mixture of luminol-S and peroxide was prepared in a 1:1 ratio and set aside. The prepared samples, antibodies, and related reagents were loaded onto the assay plate and centrifuged at 2500 rpm for 5 min at room temperature. The capillary immunoassay instrument was then initiated to analyze the samples. Data were obtained from Compass for SW software (compass 6.3.0). Subsequently, the raw data were organized for further analysis.

### 2.7. The UPLC-MS Method

Sample extraction: Six hours after microwave radiation exposure, 100 mg of rat hippocampal tissue was taken on ice, ground in liquid nitrogen, added to 3 times the volume of lysis buffer, homogenized using a vortex oscillator, and ground for 4 min with an ultrasonic cell disruptor at 45 Hz and processed for 5 min with ultrasound. After standing at −20 °C for 30 min, the supernatant was collected after centrifugation at 14,000× *g* for 30 min in a low-temperature centrifuge and filtered with a 0.45 μm filter membrane, and 20 μL of the supernatant was taken for detection in the sample bottle. An equal amount of supernatant was taken from all samples and mixed to form a quality control sample for detection.

Metabolite analysis: Samples were analyzed using a high-performance liquid chromatography–mass spectrometry system. A chromatographic column was used, with mobile phase A as acetonitrile/water and mobile phase B as isopropanol/acetonitrile, both containing 0.1% formic acid and 10 mmol/L formic acid ammonium. Sample bottles and bottle caps were used for sandwich sampling, with a sample volume of 5 μL, a column temperature of 55 °C, and a gradient elution program as follows: 0~5 min, B phase maintained at 98%; 5~8 min, B phase maintained at 30%; 8~16 min, B phase maintained at 0%; 16~20 min, B phase maintained at 98%.

Data processing: Progenesis QI 2.3 software was used to convert the raw files obtained from mass spectrometry detection into statistical data forms. Differential metabolites were collected based on the Metabolite Link database, Human Metabolome Database, and ChemSpider database to explore the relationship between differential metabolites and hippocampal function and regulation.

### 2.8. Statistical Analysis

IBM SPSS Statistics 25 software was used for statistical analysis of behavioral results and quantitative results of neuronal ultrastructure. Data are expressed as mean ± standard error ($\bar{x}\pm s$), and independent sample *t*-tests were used for statistical analysis, with *p* < 0.05 indicating statistical significance. Data plotting was performed using GraphPad Prism 8.

## 3. Results

### 3.1. Microwave Radiation Causes the Memory and Spatial Exploration Abilities in Rats to Decline 

**Figure 2:**
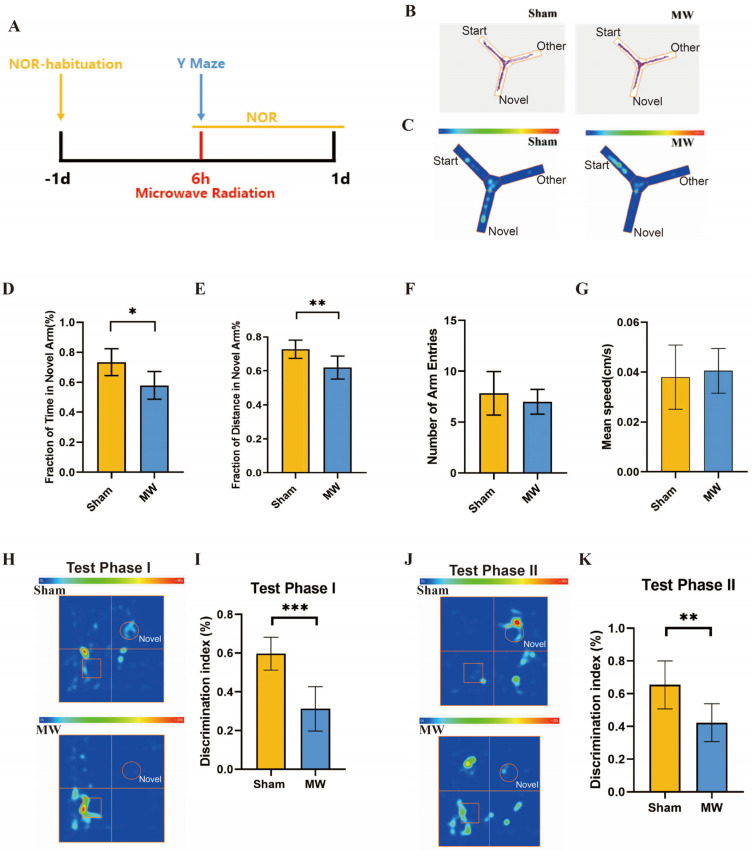
Changes in memory and spatial exploration ability of rats after microwave radiation. (**A**) Experimental timeline of the Y-maze test and NOR test. (**B**) Rat movement trajectory plot. (**C**) Heat map of rat movement trajectory. (**D**) Exploration time of novel arm. (**E**) Exploration distance of novel arm. (**F**) Number of entries into novel arm. (**G**)Average speed of rat. (**H**) Heat map of rat movement trajectory in Test Phase I, box represent rectangular prism (old object), and circle represent cone (new object). (**I**) DI of rats in Test Phase I. (**J**) Heat map of rat movement trajectory in Test Phase II, box represent rectangular prism (old object), and circle represent cylinder (another new object). (**K**) DI of rats in Test Phase II. * indicates *p* < 0.05, ** indicates *p* < 0.01, *** indicates *p* < 0.001 compared to the Sham group.

Firstly, we assessed the effects of microwave radiation on memory and spatial exploration abilities using behavioral experiments (Figure 2A). The Y maze is a method designed to assess spatial memory and exploratory ability in rodents, which is strongly correlated with hippocampus (HPC) synaptic plasticity [21,24]. Y-maze results (Figure 2B,C) revealed that compared to the Sham group, rats in the MW group had a significant decrease in the total time spent exploring the novel arm (Figure 2D) and a significantly shortened total distance traveled in the novel arm (Figure 2E). There were no differences in the number of entries into the novel arm and average movement speed between groups (Figure 2F,G). In addition, we performed the NOR test to assess the learning and recognition memory of rats [25]. The heat map of the rats’ movement trajectory showed a decrease in the exploration frequency of new objects after microwave radiation (Figure 2H,J). We found that DI of rats in Test Phase I and Test Phase II significantly declined after microwave radiation (Figure 2I,K). These results indicate a decline in rat spatial memory, recognition memory, and spatial exploration abilities caused by microwave radiation.

### 3.2. Microwave Radiation Causes Ultrastructural Damage to Hippocampal Neurons in Rats

**Figure 3:**
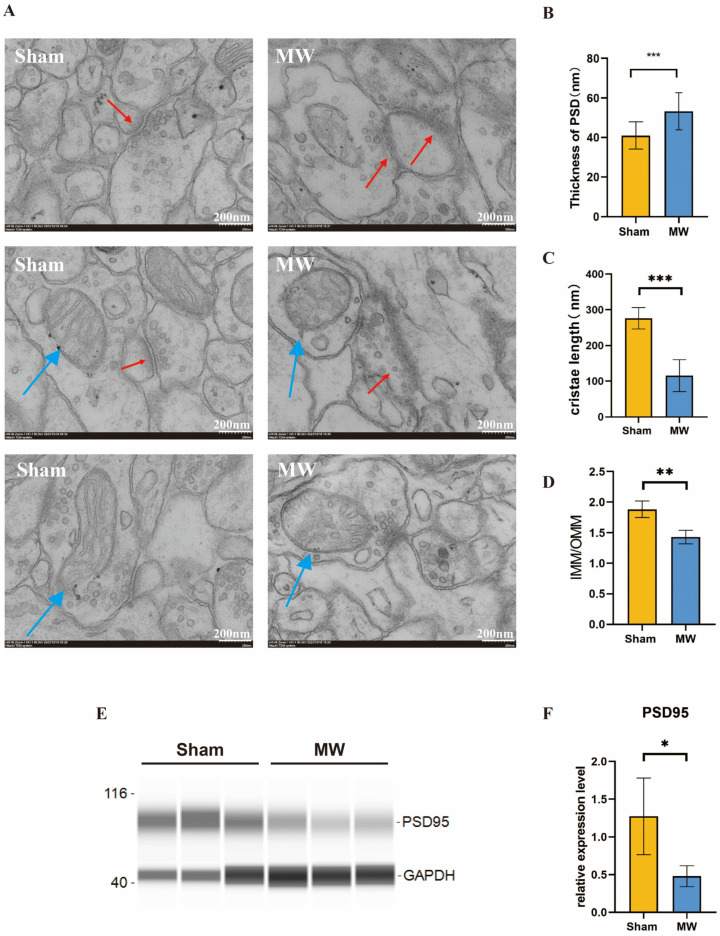
Ultrastructural changes in synaptic morphology of rat hippocampal neurons after microwave radiation. (**A**) Synaptic ultrastructure. (**B**) Quantitative analysis of postsynaptic density thickness. (**C**) Length of mitochondrial cristae. (**D**) Ratio of the mitochondrial inner membrane to outer membrane. (**E**) Representative images of PSD95 bands by Western blot. (**F**) Statistical analysis of the PSD95 level. Red arrows indicate synaptic structures, while blue arrows indicate mitochondria. * indicates *p* < 0.05, ** indicates *p* < 0.01, *** indicates *p* < 0.001 compared to the Sham group.

To further understand the changes in the ultrastructure of hippocampal neurons after microwave radiation, we observed the synaptic structure and neuronal mitochondria of the rat hippocampus through electron microscopy. We found that the synaptic clefts were blurred, the postsynaptic densities thickened, and the mitochondria exhibited fragmentation and vacuolization. (Figure 3A). Quantitative results showed that compared to the Sham group, the MW group exhibited a significant increase in postsynaptic densities (Figure 3B). In addition, Western blot was performed to assess the level of PSD95, which is a synaptic-associated protein [26]. The protein level was reduced in the MW group (Figure 3E,F), indicating damage to the synaptic plasticity of hippocampal neurons in rats [27]. The mitochondrial cristae length significantly decreased (Figure 3C), and the ratio of the inner mitochondrial membrane to the outer mitochondrial membrane (IMM/OMM) notably reduced in hippocampal neurons after microwave radiation (Figure 3D). These findings indicate that microwave radiation induces ultrastructural damage to hippocampal neurons in rats by disrupting synaptic and mitochondrial structures.

### 3.3. Microwave Radiation Causes Metabolic Profile Changes in Hippocampal Tissues

**Figure 4:**
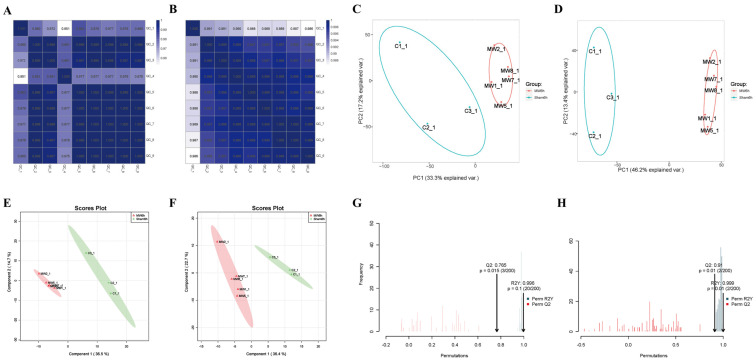
Quality control of non-targeted metabolomics in rat hippocampal tissue after microwave radiation. (**A**) Heat map of inter-sample correlation in negative ion mode QC samples. (**B**) Heat map of inter-sample correlation in positive ion mode QC samples. (**C**) PCA plot in negative ion mode. (**D**) PCA plot in positive ion mode. (**E**) PLS-DA plot in negative ion mode. In the MW group, the samples are as follows: MW1-1, MW2-1, MW5-1, MW7-1, MW8-1. (**F**) PLS-DA plot in positive ion mode. (**G**) OPLS-DA plot in negative ion mode. (**H**) OPLS-DA plot in positive ion mode.

In the correlation heat maps of quality control (QC) samples (Figure 4A,B), the correlation coefficient between QC samples was ≥0.951, indicating that the types and abundance of metabolites in QC samples were within the range of random errors. Typically, a correlation coefficient > 0.75 between QC samples is considered indicative of reliable data quality. Principal component analysis (PCA) revealed good intra-group clustering and distinct inter-group differences in both negative and positive ion modes (Figure 4C,D). Partial least-squares discriminant analysis (PLS-DA) of the metabolites between the two groups and the control group showed clear separation and significant differences in both negative and positive ion modes (Figure 4E,F). Orthogonal partial least-squares discriminant analysis (OPLS-DA) indicated excellent model fitting (R2Y = 0.996 in negative ion mode, R2Y = 0.999 in positive ion mode) and predictive ability (Q2 = 0.769 in negative ion mode, Q2 = 0.91 in positive ion mode) (Figure 4G,H), suggesting robust model performance (R2Y ≈ 1) and predictive capability (Q2 ≥ 0.4).

**Figure 5:**
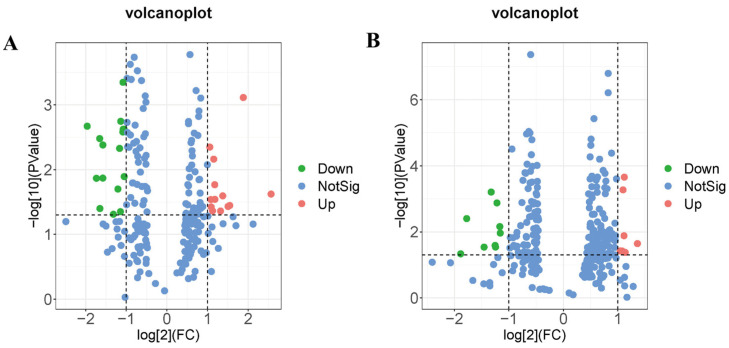
Volcano plots of differential metabolites in rat hippocampal tissue after microwave radiation. (**A**) Under negative ion mode. (**B**) Under positive ion mode.

Untargeted metabolomics analysis was conducted to analyze the metabolites of hippocampal tissue. Annotation analysis of metabolites through HMDB revealed 45 differential metabolites in the MW group compared to the Sham group. These metabolites primarily belong to categories such as lipids and lipid-like molecules, alkaloids and derivatives, benzenoids, organic acids and derivatives, organic nitrogen compounds, organic oxygen compounds, organoheterocyclic compounds, phenylpropanoids and polyketides, and eight unclassified metabolites (Figure 5, Table 1). For instance, within the category of lipids and lipid-like molecules, cholesterol sulfate, SM(d16:1/24:1(15Z)), and linoelaidylcarnitine were significantly elevated (Table 1); meanwhile, within the same category, tetrahydrocortisol was notably decreased (Table 1). Among organic acids and derivatives, L-phenylalanine showed a significant decrease (Table 1), while among organic nitrogen compounds, histamine exhibited a significant decrease (Table 1). These findings suggest that microwave radiation induces fluctuations in the metabolic profile of hippocampal tissue in rats, primarily affecting lipid metabolism pathways and metabolites related to amino acid biosynthesis pathways.

### 3.4. Metabolic Pathway Analysis of Differential Metabolites in Rat Hippocampal Tissues after Microwave Radiation

**Figure 6:**
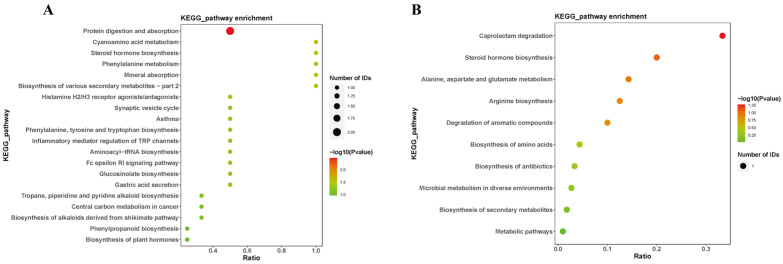
Pathways of sensitive metabolites in rat hippocampus after microwave radiation. (**A**) Under negative ion conditions. (**B**) Under positive ion conditions.

Annotation of the differential metabolites was conducted using the Kyoto Encyclopedia of Genes and Genomes (KEGG) for pathway analysis (Figure 6). The results revealed that the metabolic pathways primarily affected by differential metabolites include cholesterol sulfate and tetrahydrocortisol involved in the steroid hormone biosynthesis. L-phenylalanine participates in the phenylalanine, tyrosine, and tryptophan biosynthesis, as well as the phenylalanine metabolism. Histamine was associated with the neuroactive ligand–receptor interaction, as well as the inflammatory mediator regulation of transient receptor potential (TRP) channels. These findings suggest that microwave radiation may induce neuroinflammation, hinder neurotransmitter synthesis, and disrupt steroid hormone biosynthesis, thereby affecting the structure of hippocampal tissues and related learning and memory functions in microwave-exposed rats.

## 4. Discussion

The brain is the most sensitive target organ to microwave radiation, with the hippocampus being one of the most vulnerable brain regions. Previous studies have shown that microwave radiation can cause structural damage to hippocampal tissues and impair related cognitive functions. However, the sensitive indicators of this damage and the underlying mechanisms have not been fully elucidated.

The structure and function of the brain are highly complex, and brain cells can adapt metabolically to cope with changes in complex environmental factors. Metabolites in the brain undergo dynamic changes during development and aging [12,28]. By detecting changes in brain metabolites, it is possible to assess brain diseases and injuries, and identify differential metabolites as sensitive indicators [29]. However, the fluctuation and role of metabolites in microwave-induced learning and memory impairment are still unclear.

In this study, we first established a model of learning and memory impairment in rats and found structural damage to hippocampal tissues. Subsequently, we analyzed the metabolome of hippocampal tissues using non-targeted metabolomics. The results showed significant changes in the metabolic profile of rat hippocampal tissues after microwave radiation exposure, particularly significant increases in cholesterol sulfate, SM(d16:1/24:1(15Z)), and linoelaidylcarnitine, and significant decreases in tetrahydrocortisol, L-phenylalanine, and histamine. These metabolites are involved in processes such as steroid hormone biosynthesis, the interaction between neurotransmitters and receptors, and the inflammatory mediator regulation of TRP channels.

Steroid hormones are hormones secreted by the adrenal glands and gonads, derived from cholesterol. In addition, the brain also synthesizes neurosteroids from cholesterol [30]. They play a protective role in nerve nutrition and have important effects on cognition and memory [30]. Neurosteroids can induce neural plasticity, thereby affecting the occurrence of learning and memory. Studies have shown that estrogen, a steroid hormone, can lower the threshold for synaptic changes related to LTP by inducing excitatory postsynaptic potentials (EPSPs), which are the most relevant synaptic changes to memory formation [30,31,32]. Moreover, estrogen upregulation increases LTP magnitude induced by theta burst in male rat hippocampal slices [32,33]. Our findings suggest that the upregulation of cholesterol sulfate and downregulation of tetrahydrocortisol in hippocampal tissues after microwave radiation exposure may jointly affect the synthesis of steroid hormones. The upregulation of cholesterol sulfate, which is part of a branch of steroid hormone synthesis, suggests an increased synthesis of this branch, leading to reduced steroid hormone synthesis. This result was corroborated by the downregulation of tetrahydrocortisol. This indicates that microwave radiation may affect synaptic plasticity in the rat hippocampus by hindering the synthesis of steroid hormones, further affecting learning and memory.

With the emergence and improvement of lipidomics, sphingomyelin and ceramide have received increasing attention in neurodegenerative diseases, especially dementia and Alzheimer’s disease [33]. Levels of sphingomyelin and ceramide were found to be generally higher in the cerebrospinal fluid and autopsy results of Alzheimer’s disease patients, and the disease was more severe [34]. Our study found that levels of SM(d16:1/24:1(15Z)) increased in rat hippocampal tissues after microwave radiation exposure. This result suggested disrupted phospholipid metabolism and lipid toxicity causing damage to hippocampal tissues, resulting in cognitive impairment, and learning and memory deficits.

L-carnitine is a long-chain acylcarnitine that transports medium-chain fatty acids into mitochondria as a carrier in the beta-oxidation process, which is essential for cellular energy metabolism [35]. L-carnitine and acetyl-L-carnitine are involved in lipid metabolism and neuronal energy metabolism, regulating neurotransmitter synthesis and transmission [36,37]. In addition, L-carnitine uptake can serve as a rate-limiting step for astrocyte ketone body generation, and ketone bodies can be obtained by neighboring neurons of astrocytes as substrates for energy metabolism [37]. The increased abundance of linoelaidylcarnitine may be due to microwave radiation-induced damage to neuronal glia, leading to reduced uptake and further functional decline.

Amino acids directly or indirectly participate in many functions in the brain, and free amino acids play important roles as neurotransmitters and neuromodulators in synaptic transmission processes [38]. L-phenylalanine is a precursor to monoamine neurotransmitters, serotonin, and catecholamines, and enhances memory by improving central nervous system signal transduction [39]. Mohd Zulkifli Mustafa et al. [40] found that mice fed honey containing L-phenylalanine and tyrosine showed significantly shortened average escape latency in the Morris water maze test. Metabolomics combined with reverse transcription-polymerase chain reaction revealed that L-phenylalanine directly affected brain-derived neurotrophic factor (BDNF) and improved spatial memory in mice. Our study found that L-phenylalanine was downregulated in hippocampal tissues after microwave radiation exposure and participated in the biosynthesis of phenylalanine, tyrosine, and tryptophan, as well as the phenylalanine metabolism pathway, suggesting that microwave radiation may inhibit the synthesis of monoamine neurotransmitters and BNDF levels by downregulating phenylalanine, tyrosine, and tryptophan synthesis, ultimately leading to learning and memory deficits in rats.

Histamine is an active amine compound produced by the decarboxylation of histidine, which plays a role in regulating brain inflammation and neurogenesis [41,42,43]. Neuroinflammation is one of the causes of neurodegenerative diseases and cognitive decline. Studies have found that histamine can alleviate inflammation in the hippocampal body of mice and inhibit the death of microglia [43]. Furthermore, the lack of histamine in mice impairs long-term social recognition memory [44]. Our study found that histamine was downregulated in rat hippocampal tissues after microwave radiation exposure and participated in the interaction between neurotransmitters and receptors, as well as the regulation of inflammatory mediators on TRP channels, suggesting that microwave radiation may suppress histamine-producing neurons by downregulating histamine levels, induce hippocampal tissue inflammation, and lead to learning and memory impairments in rats.

This study has several limitations. Through untargeted metabolomics, differential metabolites can be identified, which can then be further validated through experimental verification. Combining targeted metabolomics for further sample analysis can enhance the representativeness of the identified differential metabolites. Additionally, the metabolic pathways in which these sensitive metabolites participate can serve as pivotal points for conducting thorough investigations, unveiling the mechanisms underlying metabolite actions in microwave radiation-induced injury. Furthermore, this study focuses on the role of neuronal damage mechanisms in microwave radiation-induced learning and memory impairment. However, there are other types of neural cells in brain tissue, such as astrocytes, oligodendrocytes, and microglia. Subsequent experiments could focus on changes in glial cells after microwave radiation to study cognitive impairment.

## 5. Conclusions

**Figure 7:**
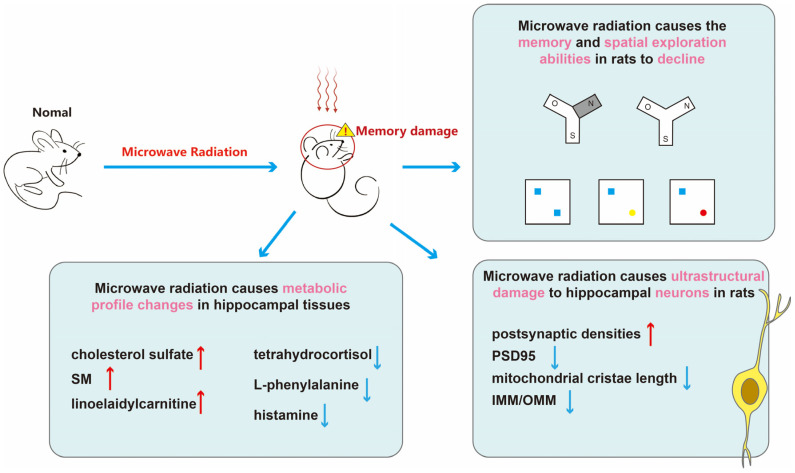
The schematic representation of this paper. First, through the Y-maze and NOR experiments, we discovered cognitive impairments in learning and memory abilities of rats after microwave radiation. Subsequently, we used transmission electron microscopy to observe the ultrastructure of hippocampal neurons and quantitatively analyzed synaptic-related proteins PSD95. By employing untargeted metabolomics, we detected differential metabolites in the hippocampal tissue, linking cognitive impairment caused by microwave radiation with sensitive metabolites. This provides sensitive indicators for brain damage caused by microwave radiation and establishes a basis for understanding its injury mechanism. In this graph, red upward arrows indicate upregulation of metabolites, while blue downward arrows indicate downregulation of metabolites, blue boxes indicate old objects, yellow circle and rad circle indicate new objects.

In summary (Figure 7), microwave radiation results in decreased learning and spatial exploration abilities in rats, structural damage to hippocampal neurons, and disruption of hippocampal tissue metabolism. Among them, cholesterol sulfate, sphingomyelin, linoelaidylcarnitine, tetrahydrocortisol, L-phenylalanine, and histamine may be sensitive metabolites for learning and memory impairment. More than half of the metabolites belong to lipids and lipid-like molecules. The content of adipose tissue in brain is second only to adipose tissue, making brain function highly sensitive to disturbances in lipid metabolism [45]. Research has shown that gene expression in adipose tissue is associated with cognitive domains, contributing to the formation of neurons and synapses [46]. Moreover, disturbances in lipid metabolism may also impact inflammatory responses and oxidative stress, thereby exacerbating cognitive decline. Dysregulation of brain lipid metabolism is implicated in the decline of cognitive function and the progression of neurodegenerative diseases, such as the generation of amyloid-beta in Alzheimer’s disease [47]. We believe that disruptions in lipid metabolism are a crucial mechanism underlying microwave radiation-induced learning and memory impairments.

Microwave technology has become an indispensable part of modern life, with humans constantly exposed to microwave radiation. In the use of wireless networks and mobile phones, some individuals may experience symptoms of mental fatigue [48]. Similarly, in laboratory research, experimental animals exposed to microwave radiation at certain power levels may develop cognitive impairments. Further research on sensitive metabolites induced by microwave radiation in the hippocampal tissue of rats, extended to the detection of metabolites in human cerebrospinal fluid and blood, can provide sensitive indicators for brain damage caused by microwave radiation and provide a basis for its injury mechanism. In addition, not only do these sensitive metabolites offer advantages in treatment, but their early detection also plays a preventive role in disease progression.

## Figures and Tables

**Table 1:** The differential metabolites in rats’ hippocampal neurons after microwave radiation.

Description	Formula	RT/min	*m*/*z*	VIP Value	*p* Value	Up or Down
***Alkaloids and derivatives***						
Tetrahydroharmol	C_12_H_14_N_2_O	4.613	405.228	2.463	0.011	Down
***Benzenoids***						
1-Hydroxypyrene glucuronide	C_22_H_18_O_7_	7.040	393.099	3.451	0.003	Down
Pentacyclo[19.3.1.1(3,7).1(9,13).1(15,19)]octacosa-1(25),3(28),4,6,9(27),10,12,15(26),16,18,21,23-dodecaene	C_28_H_24_	7.024	405.187	3.966	0.014	Down
4-Nitro-3-(trifluoromethyl)phenol	C_7_H_4_F_3_NO_3_	13.520	413.022	2.075	0.029	Up
Etiroxate	C_18_H_17_I_4_NO_4_	15.333	841.721	3.089	0.038	Up
***Lipids and lipid-like molecules***						
Cholesterol sulfate	C_27_H_46_O_4_S	8.296	931.612	2.533	0.017	Up
SM(d16:1/24:1(15Z))	C_45_H_89_N_2_O_6_P	14.087	829.640	2.238	0.029	Up
Tetrahydrocorticosterone	C_21_H_34_O_4_	7.433	373.233	4.604	0.046	Down
Linoelaidylcarnitine	C_25_H_45_NO_4_	8.328	424.341	2.416	0.001	Up
2-[Octahydro-4,7-dimethyl-1-oxocyclopenta[c]pyran-3-yl]nepetalactam	C_20_H_29_NO_3_	7.695	332.221	2.663	0.029	Down
TG(15:0/20:4(5Z,8Z,11Z,14Z)/22:6(4Z,7Z,10Z,13Z,16Z,19Z))	C_60_H_96_O_6_	11.668	935.711	3.683	0.023	Up
***Organic acids and derivatives***						
L-Phenylalanine	C_9_H_11_NO_2_	7.034	329.151	4.151	0.002	Down
Histidyltryptophan	C_17_H_19_N_5_O_3_	8.051	681.293	2.421	0.020	Down
Temocaprilat	C_21_H_24_N_2_O_5_S_2_	7.214	447.107	3.351	0.004	Down
(R)-Methylphosphonofluoridic acid 1,2,2-trimethylpropyl ester	C_7_H_16_FO_2_P	7.029	363.167	3.508	0.014	Down
Mycobactins	C_27_H_37_N_5_O_10_	7.235	590.247	10.622	0.036	Up
Argininosuccinic acid	C_10_H_18_N_4_O_6_	1.311	291.129	2.840	0.029	Down
TRIETHYL PHOSPHATE	C_6_H_15_O_4_P	6.322	183.077	2.541	0.001	Down
***Organic nitrogen compounds***						
Histamine	C_5_H_9_N_3_	7.029	221.153	2.169	0.002	Down
9-Octadecen-1-amine	C_18_H_37_N	9.889	268.299	2.660	0.013	Up
3-Cyclohexyl-1-propylsulfonic acid	C_9_H_19_NO_3_S	4.420	443.222	2.495	0.007	Down
***Organic oxygen compounds***						
Diethylpropion	C_13_H_19_NO	6.322	228.135	2.782	0.001	Down
***Organoheterocyclic compounds***						
4-Hydroxydebrisoquine	C_10_H_13_N_3_O	7.029	236.104	2.218	0.002	Down
Palonosetron	C_19_H_24_N_2_O	7.034	295.181	2.338	0.002	Down
Pyridostigmine	C_9_H_13_N_2_O_2_^+^	8.703	361.189	2.156	0.000	Down
Quinacrine	C_23_H_30_C_l_N_3_O	7.019	398.202	2.447	0.005	Down
Azaspiracid	C_47_H_71_NO_12_	11.444	886.499	2.801	0.038	Up
2,4(1H,3H)-Pyrimidinedione, 5-fluoro-1-(tetrahydro-2-furanyl)-, (R)-	C_8_H_9_FN_2_O_3_	5.178	245.059	3.796	0.001	Up
4-(Cyclohexyloxy)-2-(1-(4-[(4-methoxybenzene)sulfonyl]piperazin-1-yl)ethyl)quinazoline	C_27_H_34_N_4_O_4_S	11.489	1019.454	2.370	0.045	Down
4-Chloro-2-nitrobenzylalcohol	C_29_H_37_N_3_O_6_	7.235	568.265	7.263	0.037	Up
Ethyl 6-chlorochroman-2-carboxylate	C_12_H_13_C_l_O_3_	5.757	239.048	2.190	0.004	Up
fleroxacin	C_17_H_18_F_3_N_3_O_3_	1.288	414.127	3.949	0.040	Down
epsilon-Caprolactone	C_6_H_10_O_2_	4.978	115.075	2.141	0.038	Up
***Phenylpropanoids and polyketides***						
Diferuloylputrescine	C_24_H_28_N_2_O_6_	6.297	879.383	3.225	0.044	Up
Gonyautoxin V	C_10_H_17_N_7_O_7_S	5.214	378.085	7.283	0.024	Up
Eujambolin	C_24_H_24_O_13_	7.055	565.122	2.256	0.013	Down
Cinnamyl cinnamate	C_18_H_16_O_2_	4.470	265.121	2.366	0.042	Up
***Unclassified***						
2-Aminopropane-1,3-dithiol	C_3_H_9_NS_2_	3.534	245.029	2.600	0.025	Up
N′-(1,6-Dihydro-6-oxo-2-pyridinyl)-N,N-dipropylmethanimidamide	C_12_H_19_N_3_O	7.029	220.145	2.233	0.003	Down
N-(3-Methyl-1,1-dioxo-1,4-thiazinan-4-yl)-1-(5-nitro-2-furanyl)methanimine	C_10_H_13_N_3_O_5_S	5.089	332.057	2.600	0.049	Down
PI(20:1(11Z)/6 keto-PGF1alpha)	C_49_H_87_O_17_P	8.083	977.558	2.371	0.007	Up
Cer(d18:1/18:1(12Z)-2OH(9,10))	C_36_H_69_NO_5_	11.607	594.507	3.220	0.043	Up
Aacocf3	C_21_H_31_F_3_O	4.564	379.220	3.962	0.004	Down
(10R,13S,17S)-17-Hydroxy-13-methyl-10-[(4-methylphenyl)methyl]-17-prop-1-ynyl-2,6,7,8,12,14,15,16-octahydro-1H-cyclopenta[a]phenanthren-3-one	C_29_H_34_O_2_	4.345	437.245	2.829	0.026	Down
MPPa (Methyl pyropheophorbide-a)	C_34_H_36_N_4_O_3_	4.993	549.287	2.392	0.000	Up

## Data Availability

The data that support the findings of this study are available from the corresponding author upon reasonable request. The data are not publicly available due to privacy and ethical restrictions.
